# The heterogeneous health state profiles of high-risk healthcare utilizers and their longitudinal hospital readmission and mortality patterns

**DOI:** 10.1186/s12913-019-4769-7

**Published:** 2019-12-04

**Authors:** Shawn Choon Wee Ng, Yu Heng Kwan, Shi Yan, Chuen Seng Tan, Lian Leng Low

**Affiliations:** 1 Duke-NUS Medical School, Singapore, Singapore; 2 Program in Health Services and Systems Research, Duke-NUS Medical School, Singapore, Singapore; 3 Saw Swee Hock School of Public Health, National University of Singapore, Singapore, Singapore; 4 SingHealth Regional Health System, Singapore Health Services, Singapore, Singapore; 5 Department in Family Medicine and Continuing Care, Population Health and Integrated Care Office, Singapore General Hospital, 20 College Road, Singapore, 169856 Singapore; 6 Singhealth Duke-NUS Family Medicine Academic Clinical Program, Singapore, Singapore

**Keywords:** High-risk healthcare utilizers, Integrated care, Transitional care program, Latent class analysis, Hospital readmissions, Mortality

## Abstract

**Background:**

High-risk patients are most vulnerable during transitions of care. Due to the high burden of resource allocation for such patients, we propose that segmentation of this heterogeneous population into distinct subgroups will enable improved healthcare resource planning. In this study, we segmented a high-risk population with the aim to identify and characterize a patient subgroup with the highest 30-day and 90-day hospital readmission and mortality.

**Methods:**

We extracted data from our transitional care program (TCP), a Hospital-to-Home program launched by the Singapore Ministry of Health, from June to November 2018. Latent class analysis (LCA) was used to determine the optimal number and characteristics of latent subgroups, assessed based on model fit and clinical interpretability. Regression analysis was performed to assess the association of class membership on 30- and 90-day all-cause readmission and mortality.

**Results:**

Among 752 patients, a 3-class best fit model was selected: Class 1 “Frail, cognitively impaired and physically dependent”, Class 2 “Pre-frail, but largely physically independent” and Class 3 “Physically independent”. The 3 classes have distinct demographics, medical and socioeconomic characteristics (*p* <  0.05), 30- and 90-day readmission (*p* <  0.05) and mortality (*p* <  0.01). Class 1 patients have the highest age-adjusted 90-day readmission (OR = 2.04, 95%CI: 1.21–3.46, *p* = 0.008), 30- (OR = 6.92, 95%CI: 1.76–27.21, *p* = 0.006) and 90-day mortality (OR = 11.51, 95%CI: 4.57–29.02, *p* <  0.001).

**Conclusions:**

We identified a subgroup with the highest readmission and mortality risk amongst high-risk patients. We also found a lack of interventions in our TCP that specifically addresses increased frailty and poor cognition, which are prominent features in this subgroup. These findings will help to inform future program modifications and strengthen existing transitional healthcare structures currently utilized in this patient cohort.

## Brief summary

High-risk healthcare utilizers were segmented into 3 classes, with Class 1 “Frail, cognitively impaired and physically dependent” having the highest 90-day hospital readmission and 30- and 90-day mortality.

## Background

Globally, healthcare systems are facing the challenge of an ageing population with multiple chronic conditions [1]. These patients often experience repeated hospitalizations and are particularly vulnerable during transitions of care, resulting in significant hospital readmissions, mortality and healthcare expenditure [2, 3]. In Singapore, a multi-ethnic nation of 5.6 million people [4] with one of the most rapidly ageing population in Asia, the 30-day all-cause readmission rates in 2010 was 11.6% [5], which increased to 19.0% for patients aged 65 years and older. This is slightly lower than the 19.6% 30-day readmission rate in the United States [6]. Additionally, healthcare expenditure is predicted to exponentially rise from Singapore Dollars (SGD) $4 billion (USD $2.98 billion) in 2011 to SGD $12 billion (USD $8.94 billion) in 2020 [7]. In response to this, the Singapore Ministry of Health (MOH) launched the Hospital-to-Home (H2H) program [8], a transitional care program (TCP) that aims to improve the transition from acute care settings back into the community [9]. Likewise, similar programs emphasising population health management have emerged amongst health systems worldwide to understand the determinants of health and deliver solutions to this emerging problem [10].

However, the effectiveness of TCPs in reducing readmission and mortality has so far provided mixed results. Most programs demonstrated an improvement in patient outcomes, but a substantial number showed limited impact or worse outcomes [11–15]. A possible explanation for such inconsistency may be due to different patient subgroups with accompanying risk profiles presenting with varying responses to a standardized TCP intervention. Such a disparity is more critical in complex, high-risk patients with heterogeneous medical and socioeconomic characteristics [3], thereby limiting effectiveness of TCPs if programs do not adequately address this variance. Increasingly, medical complexity alone is found to be insufficient in explaining patterns of post-acute repeat hospitalizations [16]. Socioeconomic risk factors such as religion, health literacy, employment, and quality of family support are also important factors driving health care utilization [17–19].

A promising approach is to segment these heterogeneous populations into relatively homogenous, distinct subgroups with similar characteristics using data. A data-driven approach towards population segmentation has emerged over the years as an attractive methodology, with its ability to utilize large amounts of healthcare dataset to generate evidence-based quantitative insights into a population’s health status, thereby informing policy decisions on population health [10, 20]. To date, data-driven segmentation has not been applied to a high-risk transitional care patient population using both medical and socioeconomic determinants of health to identify specific subgroups of the population with worse outcomes than others. The identification of the highest risk population subgroup (whom often have the poorest health outcome and highest healthcare resource needs) and its associated targetable characteristics would be fundamental towards formulating intervention priorities organized around these characteristics, thereby enabling the delivery of a more effective integrated care at better value [3, 21].

In our study, we aim to segment a high-risk patient population in the H2H program into classes of unique disease profiles and identify a patient subgroup that has the poorest 30- and 90-day hospital readmissions and mortality. In addition, we aim to describe the characteristics representing the disease profile of this segment that may account for their poor health outcomes.

## Methods

### Study site, data sources

Healthcare in Singapore is largely under the responsibility of the Singapore MOH, which uses a mixed financing system that includes nationalized healthcare insurance schemes and deductions from the compulsory savings plan Central Provident Fund (CPF), for Singapore citizens and permanent residents [22, 23]. In 2017, Singapore MOH launched the H2H program with the aim of reducing unnecessary hospital admission & utilizations [9]. It involves inpatient care coordination and community care navigation by nurses through follow-up calls and home visits for high-risk patients with complex chronic disease to ensure care continuity during the transitional period after hospital discharge. These nurses are full-time, degree-holders with an average of 5 years’ experience in hospital and/or home care in adults, supervised by masters prepared Advanced Practice Nurses. Hospital readmissions and mortality were obtained at 30- and 90-days after discharge. Duration of intervention typically lasts 6 months.

We used routinely collected clinical data from the H2H program. Data included demographics (age, gender and race), medical and socioeconomic characteristics. Based on the commonly accepted age to define an older person [24], age was dichotomized into 2 groups, ≤65 years and > 65 years. We included all H2H enrolled adult patients (21 years of age and above) who are Singaporean residents or permanent residents. The Singapore General Hospital (SGH) Population Health and Integrated Care Office approved the usage of collected data for this study. The Centralized Institutional Review Board (iSHaRe Ref. No. 201707–00005) approved this study for ethics. This study has been published as an abstract for the Society for Academic Primary Care 48th Annual Scientific Meeting conference [25].

### Variables used in the latent class analysis

Multiple variables straddling medical and socioeconomic conditions were reviewed for inclusion. Importantly, these variables were routinely collected in the program as part of patient assessment.

#### Medical characteristics

Five variables related to disease state, cognition and functional status were utilised - Charlson Comorbidity Index (CCI), Abbreviated Mental Test (AMT), Clinical Frailty Score (CFS), clinical insight [26] and Activities of Daily Living (ADLs) dependency. Clinical insight was assessed by the nurses on the presence or absence of the patients’ understanding of their own medical condition. These variables and their grouped categorical scores have been validated across several countries, including locally as a good discriminative tool for predicting disease status and health outcomes [27–31].

#### Socioeconomic characteristics

Four variables - Religion, medicine consolidation issues, quality of family support and employment - were utilized. Religion (or a professed faith) was assessed by the presence or absence of it. Medicine consolidation issues were assessed by the presence or absence of the 5 rights of medication administration: Right patient, drug, dose, route and time [32]. Quality of family support was categorised into 4 groups: absent (patient has no kin), dysfunctional (presence of a high degree of conflict, misbehaviour, neglect and/or abuse occurring continuously and regularly), distant (presence of kin but minimal contact) and supportive. Employment was categorised into 3 groups: unemployed, retired or employed.

### Latent class analysis

LCA is a data-driven method utilizing individual level observable data (indicator variables) to identify underlying latent groups of individuals (classes) [33]. Examples of successful LCA utilization in population segmentation has been demonstrated by Low et al. in the Singapore regional health system [10] and Yan et al. in a primary care population respectively [34]. In this study, 9 identified medical and socioeconomic variables were selected and described above. MPlus Version 8.2 statistical modelling software was used to perform the LCA [35].

The optimal number of classes is determined by the fit statistics and clinical interpretability. Model fit was evaluated using the Bayesian Information Criterion (BIC) and sample-size adjusted BIC (ABIC) [36]. Starting with 1 class, a lower value from BIC or ABIC from each successive model, which has one more class than the prior model, indicates a better fit. Additionally, the estimated probabilities of each indicator variables within each class provide information that describes the classes and determines whether the classes are distinct from one another and clinically interpretable. Separate LCA models were generated successively from 1 through 4 class solutions. From the LCA that corresponded to the optimal number of classes identified, the posterior probability of membership for each class is computed for each subject which is assigned to the class with the maximum posterior probability.

### Statistical analysis

Firstly, to examine whether significant differences between demographics and disease patterns exist across the classes, we used Fisher exact test for the categorical variables. Next, we identified potentially confounding factors through a univariate analysis of demographics against class and health outcomes. Lastly, to assess the association of class membership on hospital readmissions and mortality, we used logistic regression with Class 3 as reference. The models were adjusted for age. Analyses were performed using SAS, version 0.4 (SAS Institute, Inc., Cary, NC).

## Results

### Segmentation outcome

A final LCA model of 3 classes was identified based on its better statistical fit (lowest BIC and ABIC) and clinical interpretability (Additional file 1: Table S1). 752 patients enrolled from June to November 2018 were segmented into 3 classes and labelled based on the estimated probability of each indicator variable within each class: Class 1 “Frail, cognitively impaired, physically dependent”; Class 2 “Pre-frail but largely physically independent”; and Class 3 “Physically independent”. A summary of the overall prevalence of the 9 indicator variables and the percentage of individuals in each class for each variable is provided in Table 1.

**Table 1:** Demographics, indicator variables and health outcomes by class (*N* = 752)

	Total (*N* = 752)	Class 1: Frail, cognitively impaired, physically dependent (*N* = 103, 13.7%)	Class 2: Pre-frail but largely physically independent (*N* = 323, 43%)	Class 3: Physically independent (*N* = 326, 43.3%)	*P*-value
Demographics					
Age (year)					< 0.001
> 65 years	511 (68)	93 (90.3)	271 (83.9)	147 (45.1)	
≤ 65 years	241 (32)	10 (9.7)	52 (16.1)	179 (54.9)	
Range	20, 98	30, 98	35, 97	20, 95	
Gender, n (%)					0.018
Female	394 (52.4)	66 (64.1)	171 (52.9)	157 (48.2)	
Male	358 (47.6)	37 (35.9)	152 (47.1)	169 (51.8)	
Race, n (%)					0.039
Chinese	588 (78.2)	78 (75.7)	268 (83.0)	242 (74.2)	
Indian	69 (9.2)	10 (9.7)	28 (8.7)	31 (9.5)	
Malay	77 (10.2)	10 (9.7)	24 (7.4)	43 (13.2)	
Others	18 (2.4)	5 (4.9)	3 (0.9)	10 (3.1)	
Indicator variables					
Religion, n (%)					< 0.001
No	234 (31.1)	31 (30.1)	76 (23.5)	127 (39.0)	
Yes	518 (68.9)	72 (69.9)	247 (76.5)	199 (61.0)	
Medicine consolidation issues, n (%)					0.006
No	673 (89.5)	89 (86.4)	279 (86.4)	305 (93.6)	
Yes	79 (10.5)	14 (13.6)	44 (13.6)	21 (6.4)	
Quality of family support, n (%)					< 0.001
Absent	48 (6.4)	2 (1.9)	11 (3.4)	35 (10.7)	
Dysfunctional	11 (1.5)	1 (1.0)	9 (2.8)	1 (0.3)	
Distant	35 (4.7)	2 (1.9)	18 (5.6)	15 (4.6)	
Supportive	658 (87.5)	98 (95.1)	285 (88.2)	275 (84.4)	
Employment, n (%)					< 0.001
Unemployed	537 (71.4)	95 (92.2)	307 (95.0)	135 (41.4)	
Retired	30 (4.0)	5 (4.9)	9 (2.8)	16 (4.9)	
Employed	185 (24.6)	3 (2.9)	7 (2.2)	175 (53.7)	
Clinical insight, n (%)					0.003
No	45 (6.0)	5 (4.9)	30 (9.3)	10 (3.1)	
Yes	707 (94.0)	98 (95.1)	293 (90.7)	316 (96.9)	
ADL, n (%)					< 0.001
Dependent	46 (6.1)	46 (44.7)	0	0	
Moderate Assist	56 (7.4)	46 (44.7)	10 (3.1)	0	
Minimal Assist	121 (16.1)	11 (10.7)	108 (33.4)	2 (0.6)	
Independent	529 (70.3)	000	205 (63.5)	324 (99.4)	
AMT, n (%)					< 0.001
0–6 point(s)	136 (18.1)	79 (76.7)	52 (16.1)	5 (1.5)	
7–10 points	616 (81.9)	24 (23.3)	271 (83.9)	321 (98.5)	
CFS, n (%)					< 0.001
1–3 point(s)	322 (42.8)	0	27 (8.4)	295 (90.5)	
4–5 points	292 (38.8)	9 (8.7)	253 (78.3)	30 (9.2)	
6–8 points	138 (18.4)	94 (91.3)	43 (13.3)	1 (0.3)	
CCI score, n (%)					< 0.001
0 points	39 (5.2)	2 (1.9)	0	37 (11.3)	
1 point	53 (7.0)	3 (2.9)	0	50 (15.3)	
2 points	63 (8.4)	1 (1.0)	10 (3.1)	52 (16.0)	
≥ 3 points	597 (79.4)	97 (94.2)	313 (96.9)	187 (57.4)	
Health outcomes					
Hospital readmission n (%)					
30 days	112 (14.9)	18 (17.5)	59 (18.3)	35 (10.7)	0.027
90 days	182 (24.2)	35 (34.0)	86 (26.6)	61 (18.7)	0.004
Mortality, n (%)					
30 days	22 (2.9)	7 (6.8)	12 (3.7)	3 (0.9)	0.005
90 days	56 (7.4)	22 (21.4)	27 (8.4)	7 (2.1)	< 0.001

*Abbreviations – ADL* Activities of Daily Living, *AMT* Abbreviated Mental Test, *CFS* Clinical Frailty Score, *CCI* Charlson Comorbidity Index

The 3 classes displayed significantly different medical and socioeconomic characteristics (Table 1). Overall, Class 1 fared the worst in these variables: unemployment (92.2%), ADLs dependent (44.7%), poor cognition (76.7% with AMT 0–6), moderately frail (91.3% with CFS 6–8 points) and significant comorbidity (94.2% with CCI ≥3).

### Demographics

As shown in Table 1, 68% of the study population were > 65 years old. Subjects in Class 1 were the oldest (90.3% aged > 65 years old) and those in Class 3 were the youngest (45.1% aged > 65 years old) among the three classes. The majority of the study population were Chinese, paralleling the general Singapore population [37]. The differences in age, gender and race were statistically significant (*p* <  0.05).

### Hospital readmissions and mortality patterns

Table 1 shows the hospital readmissions and mortality patterns at 30 and 90 days after discharge across the 3 classes. Class 1 had the highest 90-day hospital readmission (*p* = 0.004) and 30- and 90-day mortality (*p* = 0.005 and <  0.001 respectively).

In Table 2, the segmented classes persisted to be significantly associated with 90-day hospital readmission, and 30- and 90-day mortality after adjustment for age. Class 1 had the highest odds for 90-day readmission (OR = 2.04, 95% CI: 1.21–3.46, *p*-value = 0.008), 30-day mortality (OR = 6.92, 95% CI: 1.76–27.21, *p*-value = 0.006) and 90-day mortality (OR = 11.51, 95%CI: 4.57–29.02, *p*-value< 0.001) among the 3 classes. Though not as high as Class 1, Class 2 also had higher odds for 90-day readmission (OR = 1.43, 95% CI: 0.96–2.14, *p*-value = 0.079), 30-day mortality (OR = 3.56, 95% CI: 1.02–12.43, *p*-value = 0.046) and 90-day mortality (OR = 3.84, 95% CI: 1.61–9.12, *p*-value = 0.002) when compared to Class 3.

**Table 2:** Univariate and multivariate analysis on hospital readmissions and mortality

	Unadjusted OR (95% CI)	*p*-value	Adjusted OR^a^ (95% CI)	*p*-value
Hospital readmissions at 30 days		0.029^*^		0.103^*^
Class 1: Frail, cognitively impaired and physically dependent	1.75 (0.95, 3.23)	0.075	1.61 (0.84, 3.08)	0.153
Class 2: Pre-frail, but largely physically independent	1.80 (1.15, 2.81)	0.011	1.67 (1.03, 2.71)	0.037
Class 3: Physically independent	*Reference*			
Hospital readmissions at 90 days		0.005^*^		0.025^*^
Class 1: Frail, cognitively impaired and physically dependent	2.20 (1.34, 3.61)	0.002	2.04 (1.21, 3.46)	0.008
Class 2: Pre-frail, but largely physically independent	1.53 (1.05, 2.21)	0.026	1.43 (0.96, 2.14)	0.079
Class 3: Physically independent	*Reference*			
Mortality at 30 days		0.012^*^		0.021^*^
Class 1: Frail, cognitively impaired and physically dependent	7.06 (1.94, 25.71)	0.003	6.92 (1.76, 27.21)	0.006
Class 2: Pre-frail, but largely physically independent	3.61 (1.09, 11.94)	0.036	3.56 (1.02, 12.43)	0.046
Class 3: Physically independent	*Reference*			
Mortality at 90 days		< 0.001^*^		< 0.001^*^
Class 1: Frail, cognitively impaired and physically dependent	11.57 (4.88, 27.46)	<.001	11.51 (4.57, 29.02)	< 0.001
Class 2: Pre-frail, but largely physically independent	3.84 (1.68, 8.76)	0.001	3.84 (1.61, 9.12)	0.002
Class 3: Physically independent	*Reference*			

^a^Outcomes are adjusted by age. ^*^P-value for the class variable

## Discussion

Utilizing LCA, this study segmented the heterogeneous health profiles of H2H patients into 3 classes with distinct medical, social and demographic patterns. We further showed the different health outcomes between the 3 classes, demonstrating the utility of population segmentation in prognosticating patients and highlighting cohorts of patients that require differing tiers of care. Such an understanding would be fundamental for health policymakers and clinicians to make informed decisions on targeted health interventions for each class, allowing for optimal resource allocation and better health outcomes in a resource-strapped environment.

Class 1 “Frail, cognitively impaired, physically dependent” is prominent with the highest 90-day readmission and 30- and 90-day mortality amongst the three classes.. These highlights important areas of intervention for this high risk class in the existing H2H program, namely interventions targeting frailty and cognition. Our findings are congruent with existing literature, where patients who are frail and/or have poor cognition have poorer health outcome and increased healthcare utilization [38, 39]. In addition, the lack of specific clinical pathways addressing cognition is also echoed by previously conducted systematic reviews on TCPs and its components globally [40, 41], highlighting the need for the development of more robust interventions in TCPs targeting these deficits.

In terms of frailty, multicomponent interventions spanning multiple domains such as physical exercise programs (such as Tai Chi and resistance training), cognitive training, and provision of nutritional supplements have been found to be consistently successful and may be incorporated in TCPs as deemed appropriate [42]. With regards to cognition, specifically dementia, non-pharmacological interventions that are low-cost and safe such as cognitive stimulation therapy and reality orientation have been found to be correlated with cognitive and behavioural benefits [43]. Additionally, given that caregiver burden factors in as a major component affecting dementia patients’ outcome, adequate interventions such as caregiver training and education, and peripheral supportive infrastructures are necessary to address this deficit [44]. A Alternatively, given the high mortality rate for Class 1, palliative care may be a viable option to provide for end-of-life needs; seeking to improve quality of life and potentially reduce healthcare utilisation [45], especially when the extension of life may be futile or at the expanse of patients’ overall well-being. Indicators such as Advance Care Planning and appropriate place of death may provide insights into a TCP’s effectiveness, in addition to metrics such as readmission and mortality rate.

This study had several limitations. Firstly, the unique socioeconomic characteristics of our patient population in a multi-ethnic nation Singapore meant its generalizability to other patient populations may be limited. However, given that the bulk of the class differences lie in medical factors, this limitation would be limited. Secondly, the indicators used for segmentation were routinely collected medical and socioeconomic variables, which may not capture all health determinants. Future research may expand routinely collected data to variables showing a major influence on health outcomes, such as mental health and substance abuse [16]. Thirdly, the small Class 1 sample size (*N* = 103) may have contributed to the lack of significance for Class 1 30-day readmission. Future research needs to be done with larger sample sizes to elicit the significance or lack thereof between class and 30-day readmission rates. Lastly, the association between class and health outcomes were not adjusted by gender and race. Although both gender and race were significantly associated with the class (exposure), they demonstrated poor association with readmissions and mortality (outcomes) (Additional file 1: Table S2). Hence, they were not regarded as true confounding factors to be adjusted for [46].

## Conclusions

We identified a high-risk patient population subgroup in the H2H program that is frail, cognitively impaired and physically dependent and has the highest overall hospital readmission and mortality risk. Interventions targeting frailty and poor cognition may be useful in this patient segment to improve health outcomes. Segmentation using medical and socioeconomic factors may be replicated by other health systems, forming the foundation for population-level health resource planning and tailored transitional care interventions.

## Supplementary information

**Additional file 1: Table S1.** Criteria to assess model fit for latent class analysis models. **Table S2.** Univariate analysis of demographics on health outcomes.

## Data Availability

The relevant anonymized datasets used and/or analysed during the current study are available from the corresponding author on reasonable request.

## Abbreviations

H2H: Hospital-to-home
LCA: Latent class analysis
TCP: Transitional care program
