# Supplementary material for: The heterogeneous health state profiles of high-risk healthcare utilizers and their longitudinal hospital readmission and mortality patterns
Source: BMC Health Serv Res. 2019 Dec 4;19:931. doi: 10.1186/s12913-019-4769-7 (PMC6894210; doi:10.1186/s12913-019-4769-7)
Supplement: Supplementary file 1 — Additional file 1: Table S1. Criteria to assess model fit for latent class analysis models. Table S2. Univariate analysis of demographics on health outcomes. [file 12913_2019_4769_MOESM1_ESM.docx]

**Additional file 1: Table S1**: Criteria to assess model fit for latent class analysis models

| Number of Classes | Class Sizes | Bayesian Information Criterion (BIC) | Sample-size adjusted BIC (ABIC) |
| --- | --- | --- | --- |
| 2 | Class 1 = 354 (47.1%)  Class 2 = 398 (52.9%) | 7838.946 | 7727.807 |
| 3 | Class 1 = 103 (13.7%)  Class 2 = 323 (43%)  Class 3 = 326 (43.4%) | 7706.053 | 7537.757 |
| 4 | Class 1 = 102 (13.6%)  Class 2 = 304 (40.4%)  Class 3 = 15 (2%)  Class 4 = 331 (44%) | 7774.839 | 7549.386 |

**Additional file 1: Table S2**: Univariate analysis of demographics on health outcomes

|  | 30-day Readmission | | 90-day Readmission | | 30-day Mortality | | 90-day Mortality | |
| --- | --- | --- | --- | --- | --- | --- | --- | --- |
|  | **OR (95% CI)** | **p-value** | **OR (95% CI)** | **p-value** | **OR (95% CI)** | **p-value** | **OR (95% CI)** | **p-value** |
| Age (Ref: ≤65yo) | 1.50 (0.94, 2.38) | 0.086 | 1.46 (1.00, 2.13) | 0.050 | 1.90 (0.67, 5.38) | 0.230 | 2.11 (1.06, 4.20) | 0.034 |
| Gender (Ref: Female) | 1.07 (0.71, 1.59) | 0.759 | 0.79 (0.57, 1.11) | 0.177 | 0.63 (0.27, 1.49) | 0.293 | 1.10 (0.64, 1.89) | 0.736 |
| Race (Ref: Chinese) |  | 0.518^†^ |  | 0.596^†^ |  | 0.844^†^ |  | 0.981^†^ |
| Indian | 0.91 (0.44, 1.87) | 0.787 | 1.16 (0.66, 2.04) | 0.613 | 0.68 (0.13, 3.70) | 0.656 | 0.81 (0.30, 2.21) | 0.678 |
| Malay | 1.55 (0.85, 2.86) | 0.156 | 1.44 (0.84, 2.45) | 0.185 | 1.54 (0.48, 5.00) | 0.471 | 0.94 (0.37, 2.38) | 0.902 |
| Others | 0.87 (0.22, 3.50) | 0.848 | 1.00 (0.33, 3.02) | 0.999 | 0.83 (0.04, 15.39) | 0.899 | 0.99 (0.17, 5.65) | 0.993 |

^†^Significance across all races
